# Introgression of the QTL *qSB11-1^TT^* conferring sheath blight resistance in rice (*Oryza sativa*) into an elite variety, UKMRC 2, and evaluation of its backcross-derived plants

**DOI:** 10.3389/fpls.2022.981345

**Published:** 2023-01-09

**Authors:** Md. Kamal Hossain, Mohammad Rafiqul Islam, Raman Meenakshi Sundaram, Md. Atiqur Rahman Bhuiyan, Ratnam Wickneswari

**Affiliations:** ^1^ Department of Genetics, School of Environmental and Natural Resource Sciences, Faculty of Science and Technology, Universiti Kebangsaan Malaysia, Bangi, Selangor, Malaysia; ^2^ International Rice Research Institute (IRRI), Bangladesh Country Office, Dhaka, Bangladesh; ^3^ Crop Improvement Section, Indian Council for Agricultural Research-Indian Institute of Rice Research (ICAR-IIRR), Rajendranagar, Hyderabad, India

**Keywords:** rice sheath blight, QTL-introgression, resistance, *rhizoctonia solani*, marker-assisted selection

## Abstract

**Introduction:**

Sheath blight (SB) is the most damaging fungal disease in rice caused by a soil-borne pathogenic fungus, *Rhizoctonia solani* Kuhn (R. solani). The disease resistance in rice is a complex quantitative trait controlled by a few major genes. UKMRC2 is a newly developed elite rice variety that possesses high yield potential but is susceptible to sheath blight disease indicating a huge risk of varietal promotion, mass cultivation, and large-scale adoption. The aim of our present study was the development of varietal resistance against R. solani in UKMRC2 to enhance its stability and durability in a wide range of environments and to validate the effects of an SB-resistance QTL on the new genetic background.

**Methods:**

In our study, we developed 290 BC1F1 backcross progenies from a cross between UKMRC2 and Tetep to introgress the QTL *qSBR11-1_TT_* into the UKMRC2 genetic background. Validation of the introgressed QTL region was performed via QTL analysis based on QTL-linked SSR marker genotyping and phenotyping against R. solani artificial field inoculation techniques.

**Results and Discussion:**

The QTL *qSBR11-1_TT_* was then authenticated with the results of LOD score (3.25) derived from composite interval mapping, percent phenotypic variance explained (14.6%), and additive effect (1.1) of the QTLs. The QTL region was accurately defined by a pair of flanking markers K39512 and RM7443 with a peak marker RM27360. We found that the presence of combination of alleles, RM224, RM27360 and K39512 demonstrate an improved resistance against the disease rather than any of the single allele. Thus, the presence of the QTL *qSBR11-1_TT_* has been validated and confirmed in the URMRC2 genetic background which reveals an opportunity to use the QTL linked with these resistance alleles opens an avenue to resume sheath blight resistance breeding in the future with marker-assisted selection program to boost up resistance in rice varieties.

## Introduction

Rice is the most important cereal crop where more than half of the world’s 7.8 billion population consumes rice as a staple food. More than 90% of rice consumers are in Asia including the regions of 560 million hungry people (Mohanty, 2013). Rice provides about four-fifth of the calories for more than two billion people in Asia and one-third of the calories for nearly one billion people in Africa and Latin America. However, rice production is limited by numerous biotic and abiotic constraints such as diseases and insect pests, soil salinity, drought, low soil productivity, and yield stagnation of the high-yielding rice varieties. These constraints are the main challenges to meet up the projected rice demand to feed the excess population for the next decade. Among the rice production constraints, biotic agents- fungal, bacterial, and viral diseases are the major threats.

UKMRC 2 (*Oryza sativa pv.* UKMRC 2) is a high yielding modern rice variety developed by Universiti Kebangsaan Malaysia (UKM), Bangi, Selangor, Malaysia. The variety is a crossbreed product between a modern, popular, and mega variety MR219 and a wild accession, *Oryza rufipogon*. The yield potentiality of UKMRC 2 is 10-12 tons/hectare with an average of 7.2. But the downside of the variety is, that it is susceptible to rice sheath blight (SB) disease caused by a pathogenic fungus *Rhizoctonia solani*. In Malaysia the disease is considered a major threat to rice production, therefore, the countrywide expansion of the variety is questionable due to its potential drawback to sheath blight susceptibility. Apart from the well agronomic management of the disease to overcome its limitation, genetic improvement leverages a prospective option to go ahead further. Therefore, the present study was subjective to enhance SB resistance in UKMRC 2 through QTL introgression.

Sheath blight (SB) is the most damaging fungal disease in rice caused by a soil-borne necrotrophic pathogenic fungus, *Rhizoctonia solani* Kuhn (Teleomorph: *Thanaptephorus cucumeris* (Frank Donk)) was first identified as a parasite of potato in 1898 by Khun (Almasia et al., 2008). SB in rice was first reported in 1910 in Japan (Ou, 1985). The disease is characterized by infection of plants at the late tillering stage, especially at internode elongation time. Accordingly, the disease develops an oval-shaped lesion appearing on the leaf sheath at the waterline, often at the junction of the leaf and sheath at an early stage. Gradually, lesions turn pale green to off-white with a narrow purple-brown to the brown border. At the severe stage of the disease, lesions amass each other, and the disease moves upward later leaves are attacked and irregular spots are developed. The disease spreads by soil-borne sclerotia through paddy field water. Sclerotium binds at the base of the plant and starts to proliferate. The initial infection at the waterline invades the pathogen through the hyphal invasion along the stem to the canopy which develops into water-soaked lesions, corresponding to phytotoxin-killed cells (Brooks, 2007). Under favorable conditions, crop loss can reach up to 50% (Marchetti and Bollich, 1991). Up to 40% crop loss recorded in Bangladesh (Shahjahan et al., 1986). SB is particularly severe under hot and humid climatic conditions with >80% relative humidity and 28-30^0^C air temperature. However, among the causes of disease severity, higher tiller intensity and planting density leading to a thick canopy with moist microclimates are considered the main predisposing factors favoring high yield loss (Nelson et al., 2011).

SB resistance in rice is a complex quantitative trait controlled by polygenes (Sha and Zhu, 1989; Li et al., 1995a; Pinson et al., 2005). Genetic variability in high level of SB resistance is absent in cultivated rice and its wild relatives (Bonmann, et al. 1992). Nine inbred lines derived from Pusa6B x ‘Tetep’ showed ‘moderately resistance’ to SB (Pandian et al., 2012). However, several researchers (Pan et al., 1999) proposed that SB resistance in some rice varieties is controlled by only a few major genes. Some varieties with relatively high levels of resistance to SB pathogen have been identified, such as *Tetep, Tadukan, Teqing*, Jasmine85, Pecos, Azucena, ZYQ8, Minghui63, LSBR-5, and LSBR-33 (Groth and Nowick, 1992; Li et al., 1995a; Pan et al., 1999; Han et al., 2002; Kunihiro et al., 2002; Xie et al., 1990). Rice line 32R is well documented to have high level of SB resistance derived from Nepponbare x ‘Tetep’ (Gaihre et al., 2012). Several studies reported that sheath blight resistance was controlled by a few major genes in some rice varieties (Che et al., 2002; Xiang et al., 2007). However, the advances in molecular markers have led to rapid progress in understanding the mechanisms underlying resistance to sheath blight using the quantitative trait locus (QTL) mapping method. So far, nearly 70 QTLs for sheath blight resistance have been identified in rice which is distributed on all the 12 chromosomes (Channamallikarjuna et al., 2010; Che et al., 2002; Li et al., 1995a; Han et al., 2002; Kunihiro et al., 2002; Sato et al., 2004; Pinson et al., 2005; Tan et al., 2005; Liu et al., 2009; Sharma et al., 2009; Xiang et al., 2007; Zou et al., 2000; Xie et al., 2008). The parents used for QTL mapping are limited to a few cultivars, such as Lemont, Teqing, Jasmine85, and *Tetep*. A major QTL*qSBR11-1^TT^* fine mapped in *Tetep* in 0.85 Mb region with marker interval K39512 and sbq33 (Channamallikarjuna et al., 2010). On the other hand, the greenhouse evaluation of *Tetep* against the local isolate of *R. solani* confirmed the resistance potentiality of the donor (Hossain et al., 2014). This result is very promising to utilize the QTL for breeding purposes through marker-assisted selection. Wasano (1988) developed a resistant line, 2F15-92-9-22-20-11-29 (WSS2) derived from *Tetep* crosses. Improved SB resistance has been recorded in ‘Pusa Basmati 1’ through the introgression of the major QTL *qSBR11*-*1^TT^* from *Tetep* (Singh et al., 2012). Consequently, fine mapped QTLs has been transferred into local high yielding genotypes to improve resistance (Tan et al., 2005; Zuo et al., 2008; Singh et al., 2012).

Hence, it would be advisable to initiate a breeding program through introgression of the QTL from *Tetep* into the elite genotypes, UKMRC2 for an example by marker-assisted selection of the q*SBR11-1^TT^* QTL. Therefore, the present work was focused on the development of a couple of new progenies with enhanced SB resistance as an initial step in the development of a new variety with UKMRC2 genetic background. This intended research will also open a new door of hopes for marker-assisted breeding for SB resistance in rice against the devastating pathogen *R. solani*. The present research has been concluded with an excellent output of 290 backcrossed introgression lines in which six were having a high level of resistance with the presence of the QTL q*SBR11-1^TT^*. These results were validated by QTLs analysis coupled with field phenotyping.

## Materials and methods

### Plant growth in greenhouse

Seeds of *Tetep* and UKMRC2 were germinated on moist filter paper in Petri dishes in three staggering with seven days intervals of seed placement. Fifty seeds of each entry were treated with Bavistin DF (Carbendazim0.5% w/w, BASF, Germany) for 24 hours to control seed-borne pathogens. After treatment, seeds were cleaned with distilled water several times properly and put on moist filter paper in Petri dishes. Petri dishes with about 0.5 cm water were placed on a lab bench at 30°C and incubated in 12 h dark and 12 h light conditions for seven days. Seed germination and seedling growth were monitored daily. A necessary amount of water was added to Petri dishes for the normal growth of seedlings.

A ratio of 4:1 of topsoil and cow-dung compost was filled into an autoclave bag. The soil was autoclaved at 121°C and 1.05 kg cm^-2^ for 15 min. A total of 27 (15-cm-diameter) plastic pots were filled with steam-sterilized soils, which ensured the absence of soil-borne *R. solani* inoculum. To decompose soil, pots were soaked with water for seven days. Fifty mg of Furadan 3G (Carbofuran 3% w/w) insecticides were used per pot during pot preparation to control soil-borne nematodes. Five seedlings of 7-day-old of each entry were transplanted into 25-cm-diameter prepared pots at 10 cm apart. Urea (N), triple superphosphate (P_2_O_5_), and potassium chloride (K_2_O) were applied at 850 mg, 650 mg, and 575 mg per pot, respectively. Fertilizers were applied in three equal portions during pot preparation, 21 days, and 42 days after planting. Plants were grown on the greenhouse bench until hybridization.

### Production of F_1_ generation- plant hybridization

At the flowering stage female parent with at least 70% of unanthesized spikelets were selected for hybridization. Spikelets of selected female panicles were clipped-out and anthers were removed using vacuum pump emasculator (Gast, Manufacturing Inc. MI 49023, USA) at 600 mm Ag psi. To perform artificial pollination of female parents, six to eight panicles of donor male parent were collected. In an artificial pollination chamber male panicles with dust-like pollen grains were shaken carefully to allow pollen grains to fall on the stigma of the female plants. F_1_ seed settings were monitored 5 to 7 days after pollination. Glassine bags were removed, and a seed set was observed. After at least three weeks a total of 290 F_1_ seeds were produced and mature F_1_ seeds were harvested and put into brown paper bags. Bags were labeled with the date of harvesting and cross combination. Harvested seeds were dried at 52^0^ C for 72 hours and stored at 4^0^C for further use. The F_1_ hybrids were identified and confirmed by using both phenotypic and molecular methods. BC_1_F_1_progenies were then produced following the same procedure used to produce F_1_ hybrids.

### Generation of BC_1_F_1_ backcrossed progenies

For BC1F1 progeny crossing work was repeated between F1 of UKMRC2/*Tetep* and UKMRC2. To produce BC_1_F_1_ progenies a second-round crossing was made between confirmed F_1_s and UKMRC2, as the recurrent parent. 1066 BC_1_F_1_seeds were harvested from the UKMRC2/*Tetep*//UKMRC2 crosses. After confirming the F_1_ hybrids, BC_1_F_1_progenies were produced by backcrossingUKMRC2 (UKMRC2/*Tetep*//UKMRC2). Seeds were harvested and stored after 21 to 28 days of crossing. Harvested BC_1_F_1_ seeds were then dried at 52^0^ C for 72 hours and stored at 4^0^C for further use.

### Field evaluation of BC_1_F_1_ progenies

#### Plant growing in the field

All the BC_1_F_1_and parents’ seeds were germinated in Petri dishes as per procedures used for F_1_ seeds germination. Ten-day-old 207 seedlings of the BC_1_F_1_ progenies were planted at a spacing of 20cm between rows and 15cm between plants with a single seedling per hill. Parents were transplanted after every three lines of progenies. The plot size was 2m x 3.5m. The crop was fertilized with IRRI recommended dose at 80:60:40:10 kg NPKZn and @100 kg Gypsum per hectare, respectively. Total P, K, Gypsum, and Zinc-sulphate were applied at final land preparation. Total N was applied in three equal installments at 15 days after transplanting (DAT), 45 DAT, and at the panicle initiation stage. After transplanting, the field was irrigated properly, and the required water level was maintained throughout the cropping period. A good drainage system was also maintained for the immediate release of rainwater from the experimental field during the crop growing period. Necessary intercultural operations were taken during the cropping period for proper growth and development of the plants. Weeding was done 15 days after planting with the first top dressings of N. During weeding soil crust was broken to suppress weed growth and to incorporate N fertilizer into the soil for reducing the loss of urea through denitrification. Proper control measures were taken against rice stem borer with the application of Furadan3G at one gram per square meter at tillering, heading, and panicle initiation stage.

### Plant inoculation

#### Preparation of inoculums

According to the protocol (Li, 2006) rice straw-cuttings were prepared to use as inoculums with slight modification. Rice straws were cleaned and cut into 1.0 to 1.5cm pieces. About 200 g of straw cuttings were packed into an autoclave bag and autoclaved at 121°Cfor 15min. Three-days-old fresh PDA culture of *R. solani* was used to inoculate the cuttings. Five to seven mm sized 10 to 12 PDA blocks was put into each bag. These inoculations were under aseptic conditions in a laminar airflow cabinet. Inoculated cuttings were then incubated for seven days under normal room temperature. Sixty-to-70-dayold rice plants were inoculated with straw cuttings-inoculums. Each plant was inoculated with 1.0 to 1.5 g inoculums. Inoculums were placed in between the tillers at 3 to 5 cm above the ground level of the plant. Rubber bands were used to hold the inoculums. To reveal the QTL effects, the *Rhizoctonia solani* field isolates were used to challenge the BC1F1 progenies and its parents. The anatomosis group of the fungus *R. solani* were previously classified and confirmed by Hossain et al., 2014. According to the author the protocol is in brief, the 716 bp consensus sequence of ITS-rDNA region of the pathogen was used to classify the anastomosis group. It was found that the four BLAST hits showed maximum identity of 100% with an E-value of 0.0. Our submitted sequence (GenBank Accession No.: KF312465) was found identical to the sequence, the BLAST hits of the isolates collected from rice plant, belongs to the *R. solani* anastomosis group AG1-1A.

### Disease evaluation

To evaluate the BC1F1 progenies and its parents, a field experiment was laid-out with augmented-RCB design in 5 blocks. The fixed-effect model was used to analyze the variances. After one week of inoculation, disease lesions were monitored. Data were collected after 30 days of inoculation. Days to heading, plant height, lesion height, number of panicles per hill, number of and tillers per hill were recorded according to the guidelines described in the standard evaluation system for rice (IRR, 2002). Five plants from the middle three rows of the plot from each replication were used for recording data.

#### Plant height

Plant heights were measured in centimeters from the ground level to the tip of the main tiller at maturity with the help of a meter scale.

#### Days to heading

Several days are required for 50% of the plants to show panicle emergence or blooming from the date of soaking.

#### Relative lesion height

RLH (%) was calculated using the following formula: RLH (%) = Lesion length/plant height x 100 (Sharma, 1990).

#### Disease severity index

DSI was then categorized based on RLH with a 0 to 9 scale, where 0 = no lesion and 9 = lesion visible up to the tip of the longest leaf (Prasad and Eizenga, 2008).

### SSR marker genotyping

#### DNA isolation and quantification

For SSR marker genotyping, a total of 197 leaf samples were collected. These were included 195 BC1F1 progenies and two parents. At 40-day of rice seedling age, 15 to 20 cm long second leaves were cut from five plants and put into a plastic bag. Leaf samples were stored immediately at -20^0^ C for future use. DNA was extracted following step-by-step procedures using QIAGEN DNeasy mini kit (Qiagen, Germany) as described in the manufacturer’s guidelines. Two µL of DNA was pipetted on the peddle stand of the Nanodrop-ND1000 spectrophotometer system (Thermo Scientific, USA). Absorption at 260/280 and 260/230 nm wavelengths was recorded. The purity of extracted DNA was also checked on 1% agarose gel.

#### Polymerase chain reaction

A total of 33 SSR primers were used to detect polymorphisms between parents for foreground selection. Four markers viz. RM224, K39512, RM27560, and RM7443 were identified as polymorphic (**Table 1**) between UKMRC2 and *Tetep* for the target QTL *qSBR11-1^TT^.* Each polymerase chain reaction (PCR) was conducted in total 10 µL reaction volume containing 1.0 µL 10x reaction buffer, 0.2 µL dNTPs (10 mM), 0.1µL Taq polymerase (5U/µL), 1.0 µL (5 ρM/µL) of each primer, and 1.0 µL (10 ng/µL) templates DNA and 5.7µL of ddH_2_O. The following temperature profiles and cycles were maintained using Eppendorf Nexus Gradient Thermocycler (Eppendorf, Germany): 1 cycle at 94^0^ C for 4 min followed by 35 cycles at 94^0^ C for 45 sec, annealing at 55^0^ C or 61^0^ C (depending on the primers) for 45 sec, 1 cycle at 72^0^ C for 1 min, final extension at 72^0^ C for 10 min and storage at 10^0^C

**Table 1:** Details of foreground polymorphic markers used to detect the presence of the QTL *qSBR11-1^TT^* in backcrossed introgression lines.

Locus name	Primer sequence (5’-3’)	Ann. Temp. (^0^C)	PCR product Size (bp)	
			UKMRC2	Tetep
RM224	F: ATCGATCGATCTTCACGAGG R: TGCTATAAAAGGCATTCGGG	55	150	165
K39512	F: GCCACATCAATGGCTACAACGTC R: CCAGAATTTACAGGCTCTGG	55	102	112
RM27360	F: CATGTTGCGTGTTTGTATACCACTCC R: GCCGCTGGTGAGTCGTAATGG	55	233	240
RM7443	F: ATTAACCCTTCATCAGGCTACGC R: AAAGTGCTGCGTGTTACTTTGG	55	160	172

F, forward; R, reverse.

#### PCR documentation *via* capillary electrophoresis

The primers were arranged in 6 panels consisting of all 4 dyes viz. FAM (Blue), VIC (Green), NED (Yellow), and PET (Red). The primers from the same dye were assigned in a panel with a large difference in allele size to distinguish the peak height. The forward primers were fluorescently labeled at 5’end with different dyes to incorporate into PCR. According to Zuo et al. (2011) each polymerase chain reaction (PCR) was conducted in total 20 µL reaction volume containing 2.0 µL of 10x reaction buffer (10 mM), 2.0 µL of MgCl_2_ (25 mM), 0.4 of µL dNTPs (10mM), 0.2 of µL Taq polymerase (1U), 1.0 µL of each primer (3.084 mM), and 2.0 µL (10ng/µL) templates DNA and 11.4µL of ddH_2_O. The following temperature profiles and cycles were maintained using Eppendorf Nexus Gradient Thermocycler (Eppendorf, Germany): 1 cycle at 94^0^ C for 4 min followed by 35 cycles at 94^0^ C for 45 sec, annealing at 55^0^ C or 61^0^ C (depending on the primers) for 45 sec, 1 cycle at 72^0^ C for 1 min, final extension at 72^0^ C for 10 min and storage at 10^0^C. About 1.1-1.5 µL of the amplified PCR products from each of the four fluorescent dyes were added to the mixed solution of 0.3 µL of LIZ 500 and 6.7 µL of formamide for fragment detection using capillary separation on the ABI3100 Genetic Analyzer (Applied Biosystems, USA) at FirstBase Laboratories, Seri Kembangan, Malaysia. Fragment lengths (allele size) were estimated and scored with GeneMapper version 4.0 analyzing software (Applied Biosystems, USA).

#### Gel-based detection of PCR products

To validate the results of PCR products produced by capillary electrophoresis, a gel-based detection procedures were used. According to Sambrook et al. (1989), a gel-based detection system, polyacrylamide gel electrophoresis (PAGE) was used. The procedure in brief, 50 ml of 8% gel preparation protocols - were as follows: first, a 200 ml cleaned glass beaker was poured with 26.7 ml of doubled distilled water followed by adding of 10 ml of 5% TBE solution. The mixture was then stirred and 13.3 ml of chilled acrylamide bis-acrylamide (29:1) was added very quickly. After that 300 µL of 10% ammonium persulfate (APS) was added to the beaker and mixed properly. Finally, 60 µL of TEMED was added as quickly as possible and mixed properly. The entire gel mixture was poured into a glass-plate setting and a comb was placed on the top of it. The gel was allowed to polymerize at room temperature for 60 minutes. After polymerization of the gel rubber gasket, spacers, and clamps were removed and squirted with 1% TBE solution to remove air bubbles from the wells. The gel was cast tightly on the buffer tank using two clamps. Two µL of each PCR product was mixed with 2 µL of gel loading dye. Then a total of 4 µL mixtures were pipetted into each well. After loading the PCR products, the gel was run at 100V for 2 hours using Bio-Rad PowerPac (Bio-Rad Company, Singapore). After completion of the electrophoresis, the gel was removed from the glass plates very carefully to avoid any breakage of gel. The PCR product was stained by soaking for 15 to 20 minutes in Sybr-safe gel-stain solution (10 uL/100 mL H_2_O). Subsequently, gels were visualized under a UV transilluminator, and images were documented using the Alphaimager gel documentation system (Alpha Innotech, USA).

### Statistical analysis

Average data, recorded from two parents along with their two mapping populations on plant height, days to heading, relative lesion height, and disease severity index were analyzed using R software version 4.1.2 (R Core Team, 2021) for Q-Q plots and boxplots generation. Single environment analysis with fixed effect model was used for ANOVA and phenotypic distribution analysis using PBTools 1.4. (IRRI, 2013). Genotype data of BC_1_F_1_ populations were scored as ‘A’ (AA) for parental (recurrent) type and ‘H’ (A/B) for recombinant type. QTL analyses were performed using QGene version 4.0.2 (https://www.qgene.com). Mean DSI was used for composite interval mapping (CIM) at default 15 cM fixed window with cofactor-dropping and forward regression method. The LOD threshold of ≥ 2.5 was used to declare the presence of QTLs. Additive effects and percentage of total phenotypic explained by individual QTL were estimated.

## Results

### Phenotypic performance of morphological traits of BC_1_F_1_ population

**Figure 1:**
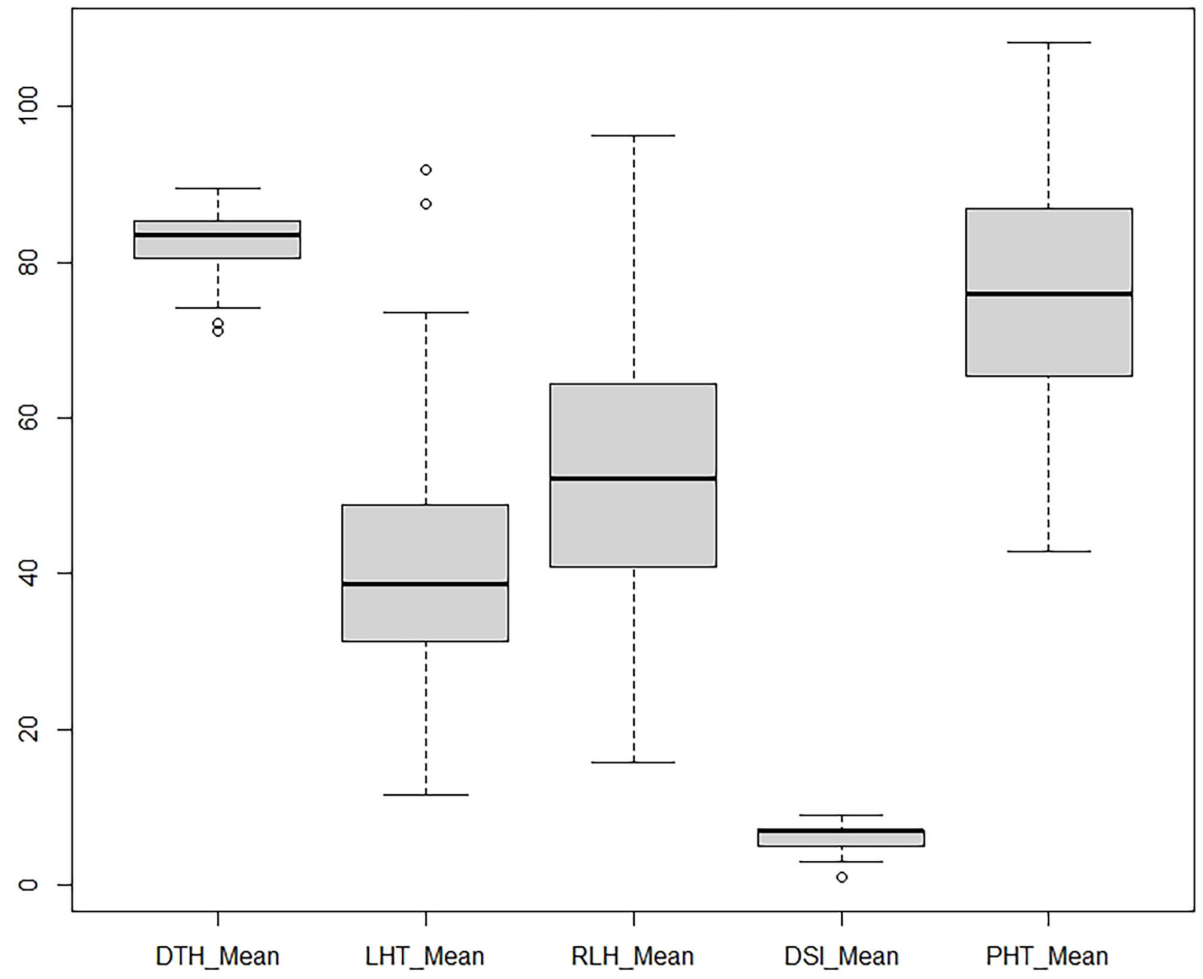
Boxplot of five morphological traits. Data were collected from 207 BC_1_F_1_ progenies from field evaluation including artificial inoculation of *R. solani isolates*. Each of the plots represents the median (dark line), 25–75 quartiles (boxes), 5th and 95th percentile values (error bars) and outliers (hollow circle). DTH, Days to fifty percnt flowering; LHT, lesion length (cm); RLH, relative lesion height (%); DSI, disease severity index (1-9 scale); PHT, plant height (cm).

**Figure 2:**
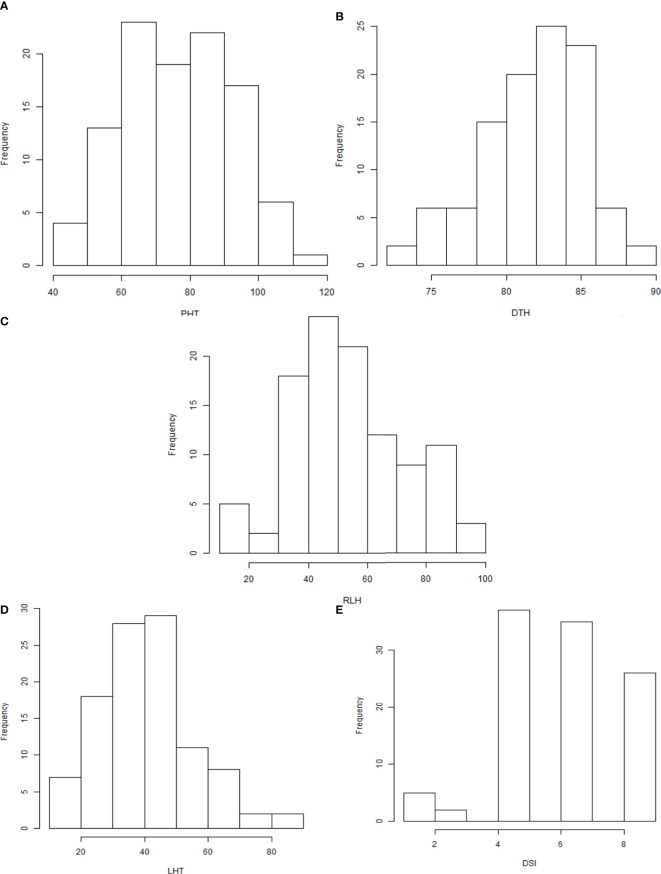
**(A–E)** Density distribution graphs of five morphological traits of 207 BC_1_F_1_ progenies. Abbreviations are mentioned in the **Figure 1**.

Observable variations in BC_1_F_1_backcross progenies were recorded in five morphological traits such as PHT, DTH, LHT, RLH, and DSI. The boxplot (**Figure 1**) showed that the *Tetep* introgression backcrossed population (BC_1_F_1_) produced wider variations in plant height ranging from 42.9 to 108.3 with a mean of 75.48 cm (**Table 2**). The average plant heights of both parents, UKMRC2 and *Tetep*, were 72.90, and 100.00 cm, respectively (**Table 2**). Phenotypic distribution analyses of plant height showed a bimodal curve in the progenies (**Figure 2A**). However, sufficient variations in days to heading were also observed in populations that ranged from 72 to 100 with a mean of 84 cm (**Table 2**). The parents UKMRC2 and *Tetep* started flowering at 92 and 83 days after planting, respectively. Distribution analysis of days to heading showed a normal curve of the progenies (**Figure 2B**). The lesion height (LHT) ranged from 10 to 70 with an average of 45 (**Figure 1**). The resistance parent *Tetep* showed the LHT at 30 cm whereas the susceptible parent UKMRC2 that of at 70 cm (**Table 2**). In the case of phenotypic distribution analysis, the graph showed asymmetric with a slight right-skewed distribution (**Figure 2C**). The BC_1_F_1_segregating populations produced adequate variations in relative lesion height (RLH) which support QTL mapping for the trait. RLH in the progeny population varied from 15.7 to 96.2% with a mean of 54.14 while in the parent it was 63.22, and 30.20% in UKMRC2 and *Tetep*, respectively. Phenotypic distribution analyses showed a normal curve for RLH (**Figure 2D**).

**Table 2:** Summary statistics of four phenotypic traits of interest for 207 BC_1_F_1_ progenies and its two parents.

Traits	Population	Range	Mean	CV	STD
PHT	BC_1_F_1_	42.9-108.3	75.48	20.12	15.18
	UKMRC2	–	72.90	8.64	6.30
	Tetep	–	100.00	11.15	11.25
DTH	BC_1_F_1_	72.0-100.0	84.03	7.26	6.10
	UKMRC2	–	91.73	1.87	1.49
	Tetep	–	82.52	2.62	2.16
RLH	BC_1_F_1_	15.7-96.2	54.14	33.34	18.05
	UKMRC2	–	63.22	28.55	18.05
	Tetep	–	30.20	33.12	10.00
DSI	BC_1_F_1_	1-9	6.45	30.48	1.97
	UKMRC2	–	7.54	20.63	1.56
	Tetep	–	3.79	48.42	1.83

CV, coefficient of variation; STD, standard deviation, other abbreviations are mentioned in the **Figure 1**.

### Phenotypic performance of morphological traits of resistant progenies

**Figure 3:**
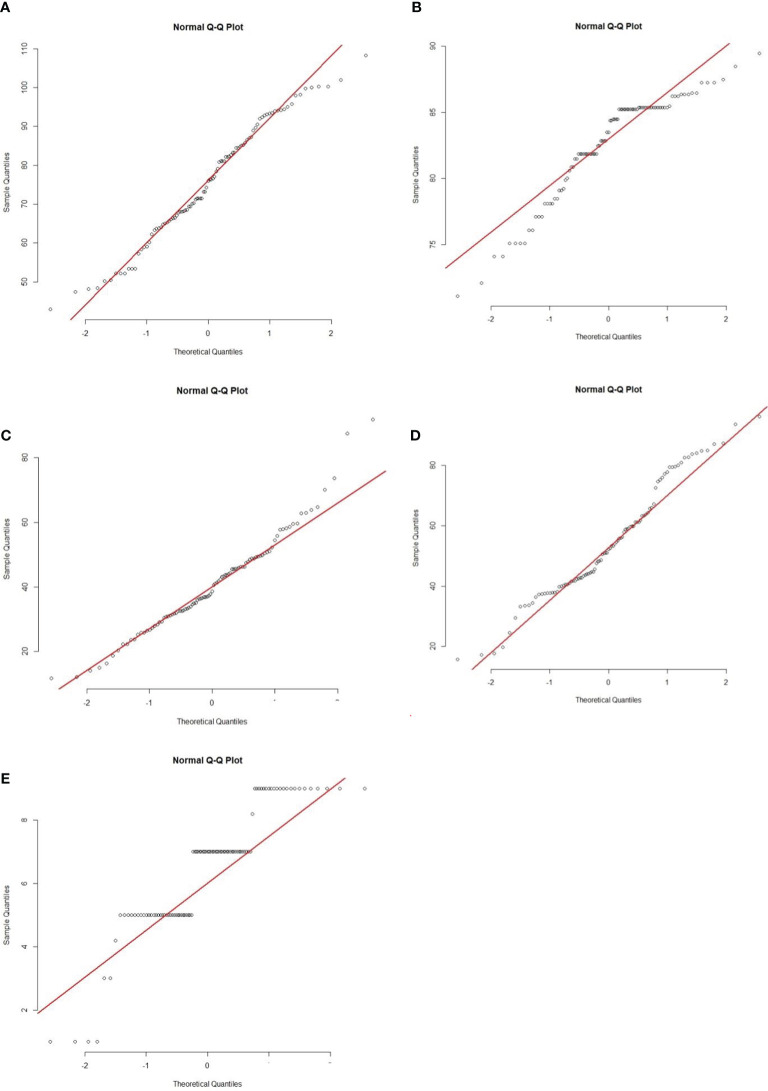
Quantile-Quantile (Q-Q) plots of five morphological traits of 207 BC_1_F_1_ progenies. **(A)** PHT, **(B)** DFF, **(C)** LTH, **(D)** RLH and **(E)** DSI. Abbreviations are mentioned in the **Figure 1**.

**Figure 4:**
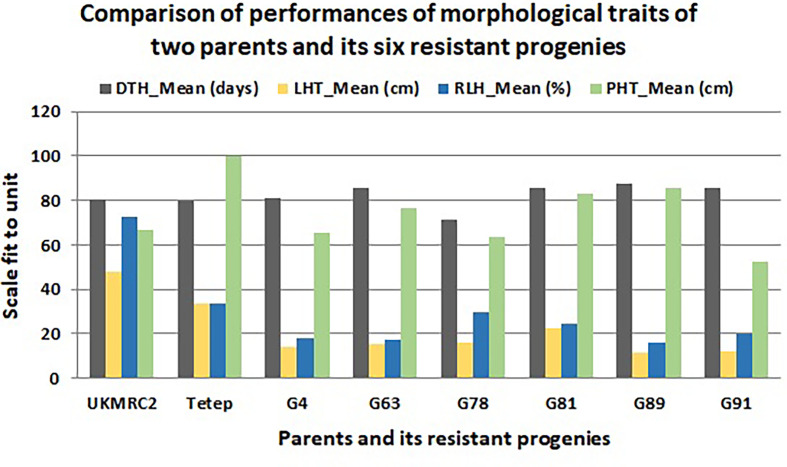
Comparison of phenotypic performance of four morphological traits of 207 BC_1_F_1_ progenies of two parents (UKMRC2 and Tetep) and six resistant BC1F1 progenies (G4, 63, 78, 81, 89, 91). Abbreviations are mentioned in the **Figure 1**.

There were some contrasting phenotypes recorded between parents and six resistant BC_1_F_1_ progenies. **Figure 3** showed closer similarity in days to flowering with a little fluctuation among the parents and progenies. The diversified characteristic in plant height morphology was found between patent and progenies, for example, in Tetep the average plant height was recorded as 100 cm. In progenies, the height was lowest in G91 (52 cm) while in G89 it was 86 cm. The Lesion height (LHT) is one of the most important morphological features in SB studies. In parents, the average 30 cm LHT was found in Tetep whereas 46 cm in UKMRC2. In the resistant progenies, the trait ranged from 10 to 20cm only (**Figure 3**). For RLH similar measurement was also recorded in parents and the progenies. In the parent, the value was approximately 30% in Tetep and 62% in UKMRC2. The lowest RLH was observed in the progeny G89 (15%) while the highest values in progenies were found at 30% in G78 (**Figure 3**). SB disease scores were evaluated with 1.0 to 9.0 DSI scales to assess the disease severity in the progenies. Six out of the 207 progenies were found under the high resistance category with DSI scores of 1.0 to 3.0 (**Figure 4**). The parent UKMRC2 showed an average DSI of 7.54 whereas *Tetep* was at 3.79.

### QTL analysis and confirmation

**Figure 5:**
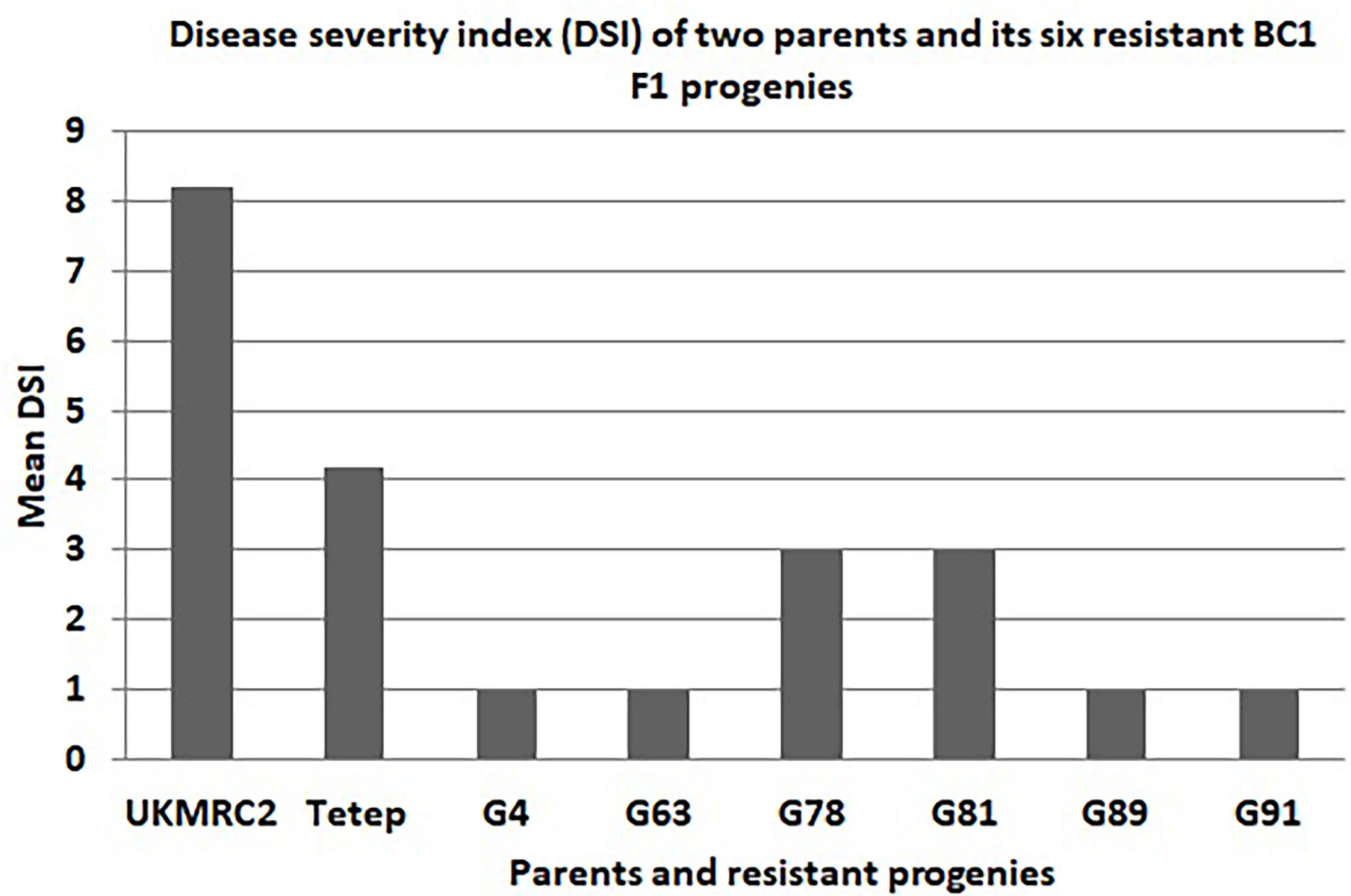
Disease severity index (DSI) graph of two parents (UKMRC2 and Tetep) and its six resistant BC_1_F_1_ progenies (G4, 63, 78, 81, 89, 91). DSI was scored on a 1-9 scale where 1 indicates highly resistant and 9 for highly susceptible disease reactions.

**Figure 6:**
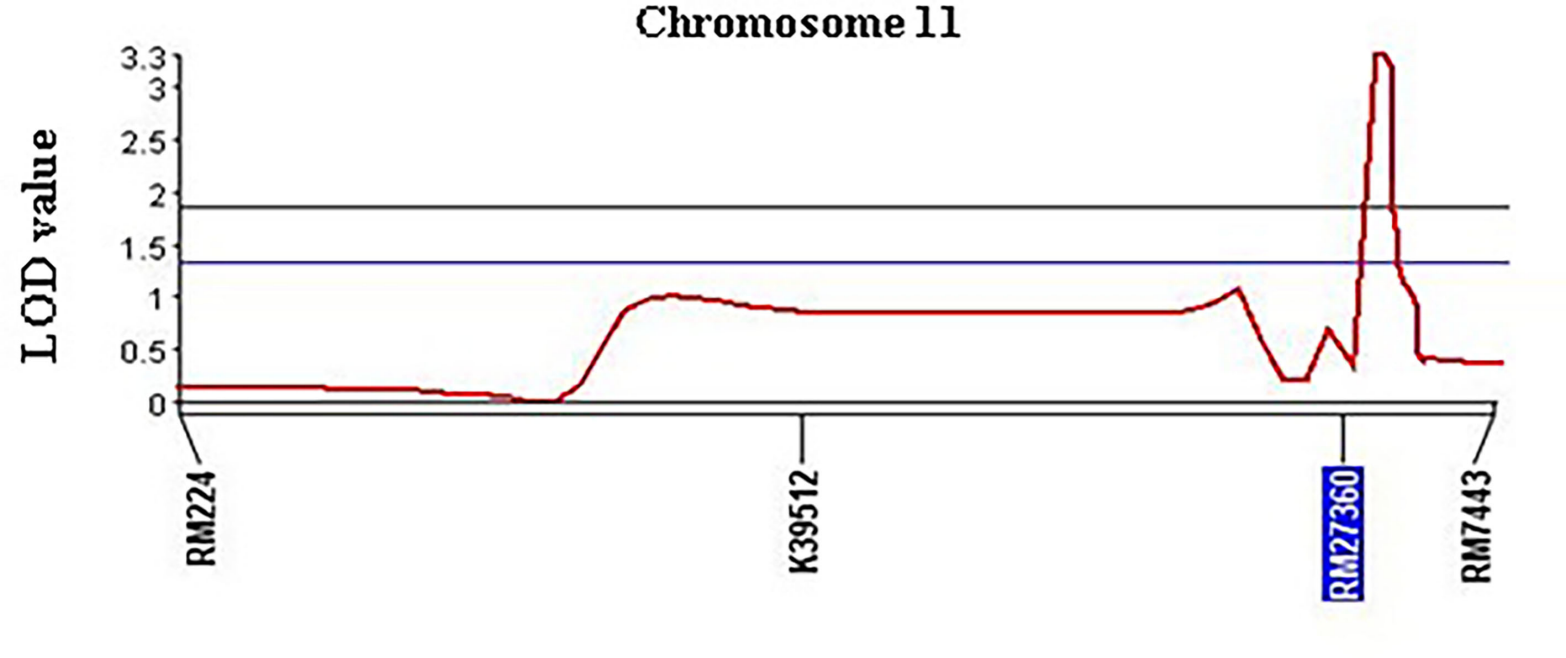
Chromosomal position and LOD scores for the QTL *qSBR11-1^TT^* on rice chromosome 11 identified by composite interval mapping (CIM) analysis of foreground SSR markers.

QTL analysis was performed using four polymorphic SSR markers’ (**Table 1**) for *qSBR11-1^TT^* using 95 BC_1_F_1_ progenies. The presence of the QTL *qSBR11-1^TT^* was detected and verified with a LOD score (3.25) using the CIM strategy (**Figure 5**). It was also estimated that the SB-resistant allele *qSBR11-1^TT^* explained a total of 14.6% phenotypic variation with an additive effect of 1.1 under field phenotyping with *R. solani* inoculation. The flanking region of the QTL was confirmed by two-interval markers, K39512 and RM7443 with a peak at RM27360 (**Figure 5**). To confirm the presence of the QTL alleles in the identified resistant progenies gel-based detection of PCR products was conducted using PAGE gel. The presence of resistance donor alleles in the identified six progenies has been confirmed by the production of PAGE-gel documentation images (**Figure 6**). The gel images of the four progenies (G4, 63, 89, 91) which showed a high-level resistance (DSI score of 1.0) contained SSR markers alleles K39512 (112bp), RM224 (165bp), and RM27360 (240bp). On the other hand, the gel image of the rest two resistant progenies (G78 and 81) (DSI score 3.0) showed the presence of the marker allele RM7443 (172bp).

## Discussion

### Breeding for sheath blight resistance in rice

Breeding for SB resistance in rice becomes difficult due to the following reasons: i) lack of complete resistance sources (Liu et al., 2006; Liu et al., 2009; Park et al., 2008), ii) accurate quantification of the disease infestation is one of the major shortcomings to identify the appropriate resistant source. Though several methods have been developed to screen resistant genotypes, most of them are tedious, time-consuming, and laborious (Park et al., 2008), and iii) SB resistance is a typical quantitative trait where many genes are involved in the resistance processes (Sha and Zhu, 1989). Consequently, it has become impossible persistently to breed the resistant genotype using conventional breeding methods. However, there are some QTLs that have been identified which show partial resistance that might facilitate molecular breeding for SB resistance. Therefore, we selected the QTL *qSBR11-1^TT^* for validation in the elite background to facilitate future marker-assisted breeding in rice to improve its resistance.

### Phenotypic distribution analysis

The BC_1_F_1_ progenies possessed significant morphological variations in PHT, DTH, LHT, RLH, and DSI revealed by distribution analysis (**Figures 2A–E**). We observed that the PHT was distributed as a bimodal pattern (**Figure 2A**) in BC_1_F_1_^TT^ which suggested that the involvement of polygenes with major genes has a large effect on controlling this agronomic trait. Sharma et al. (2009) also reported that PHT is controlled by major genes in rice while three QTLs (*QPh3a, QPh8a, and QPh9a*) controlling SB resistance have been mapped on chromosomes 3, 8, and 9 (Li et al., 1995a). On the other hand, another three different QTLs (*qPH-3, qPH-4, and qPH-11*) controlling PHT, have also been mapped on chromosomes 3, 4, and 11 (Zou et al., 2000). This distribution was clearly visualized Q-Q plot (**Figure 3A**) which revealed a normal distribution for the trait. Shapiro-Wilk test validate the findings with a significant p-value of 0.1126. DTH followed a normal distribution (**Figure 2B**) for both the populations which indicated a polygenic control of the trait. Zou et al. (2000) mapped four QTLs (*qHD-2, qHD-3, qHD-5, and qHD-7*) for DTH that support our findings. Sharma et al. (2009) reported from 2-year replicated field trials and genotyping with 149 SSR markers for SB reactions, PHT, and DTH and concluded that SB ratings were significantly correlated with PHT and heading date. However, phenotypic distribution analysis of RLH revealed a continuous distribution in the BC_1_F_1_^TT^ populations (**Figure 2**) which is expected for quantitative traits genetics. SB resistance in rice, however, is a typical quantitative trait controlled by polygenes (Sha and Zhu, 1989; Li et al., 1995b; Pan et al., 2005; Channamallikarjuna et al., 2010). DSI was used to phenotype the resistance level of the backcrossed introgression lines for detection of QTL effects and verification of the presence of the QTL*qSBR11-1^TT^*. However, the overall DSI showed a continuous distribution in the population BC_1_F_1_^TT^ (**Figure 1**) indicating a polygenic control of the trait termed as ‘quantitative resistance’. A similar type of distribution was reported by Sharma et al. (2009). The Q-Q plots for DTH, LTH, RLH and DSI (**Figures 3B–E**) indicated that the distribution for those traits were non-normal which were supported and validated by p-values of Shapiro-Wilk test. However, several researchers reported that SB resistance in rice is controlled by major as well as minor genes (**Table 3**). For example, *qSB-9^TQ^* (*Teqing*)*, qSB9-1* and *qSB9-2 (*Jasmine85)*, and qSBR11-1^TT^* (*Tetep*) have been mapped as major effects QTLs (**Table 3**) while accordingly to Li et al. (1995b) these three QTLs: *QSbr3a, QSbr8a*, and *QSbr9a* on chromosomes 3, 8, and 9 were reported as minor respectively.

**Table 3:** Details of major QTLs and their associated markers conferred sheath blight resistance in rice.

Chrm.	QTL	Linked marker	Mapping population	Reference
11	QDs11b	RM224	‘Teqing’/Tarom Molaii	Li et al., 2009
11	QRh11	RM224	‘Teqing’/Tarom Molaii	Li et al., 2009
11	QDs11a	RM187	‘Teqing’/Binam	Li et al., 2009
11	qSBR11-1	RM224	HP2216/’Tetep’	Channamallikarjuna et al., 2010
11	qSBR11-2	RM3428-RM209	HP2216/’Tetep’	Channamallikarjuna et al., 2010
11	qSBR11-3	RM536-RM202	HP2216/’Tetep’	Channamallikarjuna et al., 2010

### QTL detection and verification

The development of SB-resistant genotypes through MAS of the QTL *qSBR11-1^TT^* is feasible (Zuo et al., 2014). Moreover, the QTLs *qSBR11-1^TT^* has been mapped as a major QTLs in *Tetep* which demonstrated resistance potentialities (Liu et al., 2013). Based on the previous evidence, it is considered that the QTL *qSBR11-1^TT^* is the most potential to breed rice for SB resistance.

**Figure 7:**
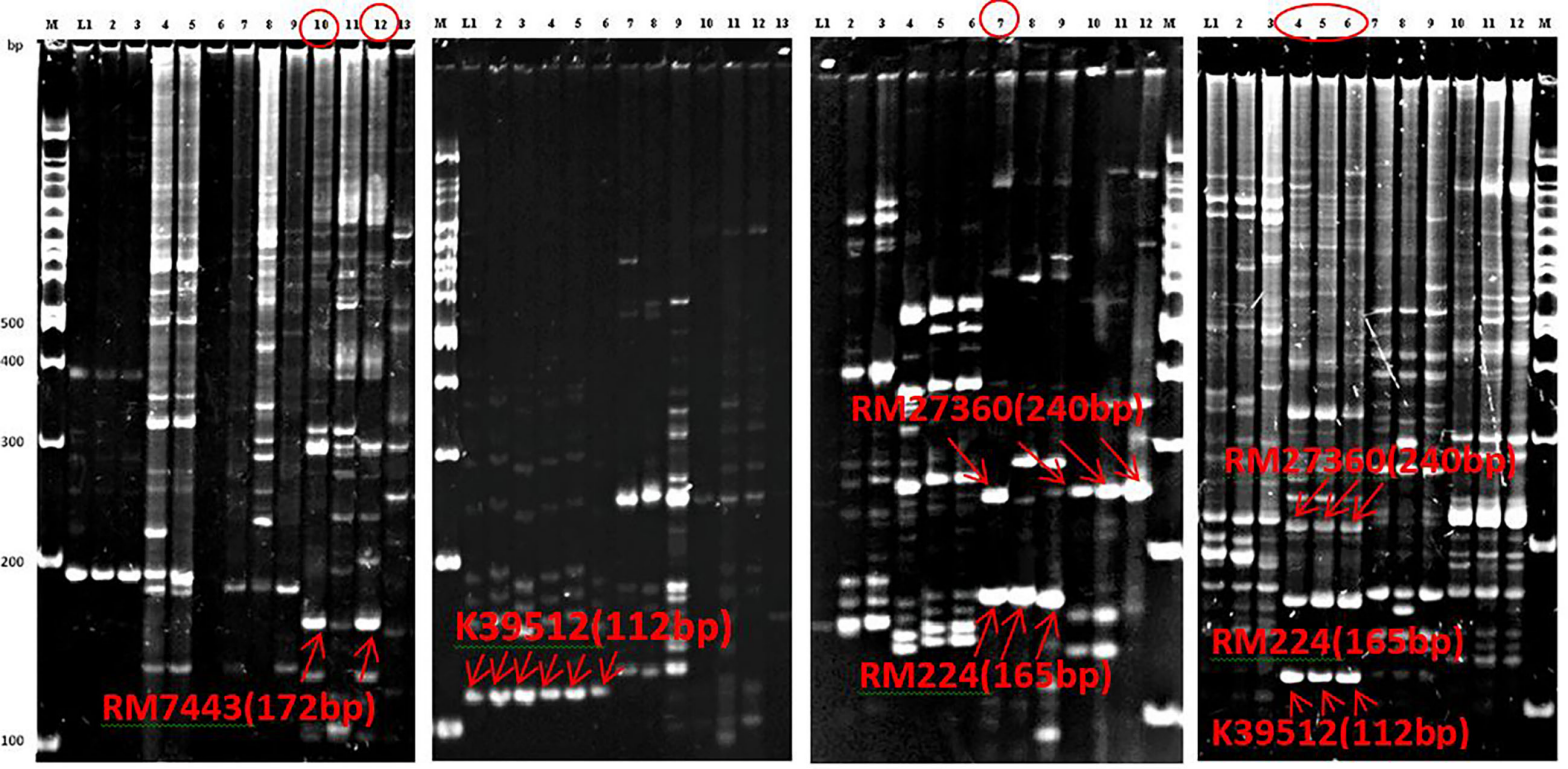
Polyacrylamide gel electrophoresis (PAGE) gel images of PCR products of the 52 identified BC1F1 progenies in which six were resistant indicated the presence of resistant donor alleles. Top-left: G78 and 81 possess RM7442 allele with DSI 3.0, Mid-left: six progenies contain K39512 allele with improved resistance not selected, Mid-right: The progeny G91 having RM27360 and RM224 alleles with DSI 1.0, and Top-right: The three resistant progenies contain the three resistant alleles, RM27360 RM224 and K39512 alleles with DSI 1.0.

**Figure 8:**
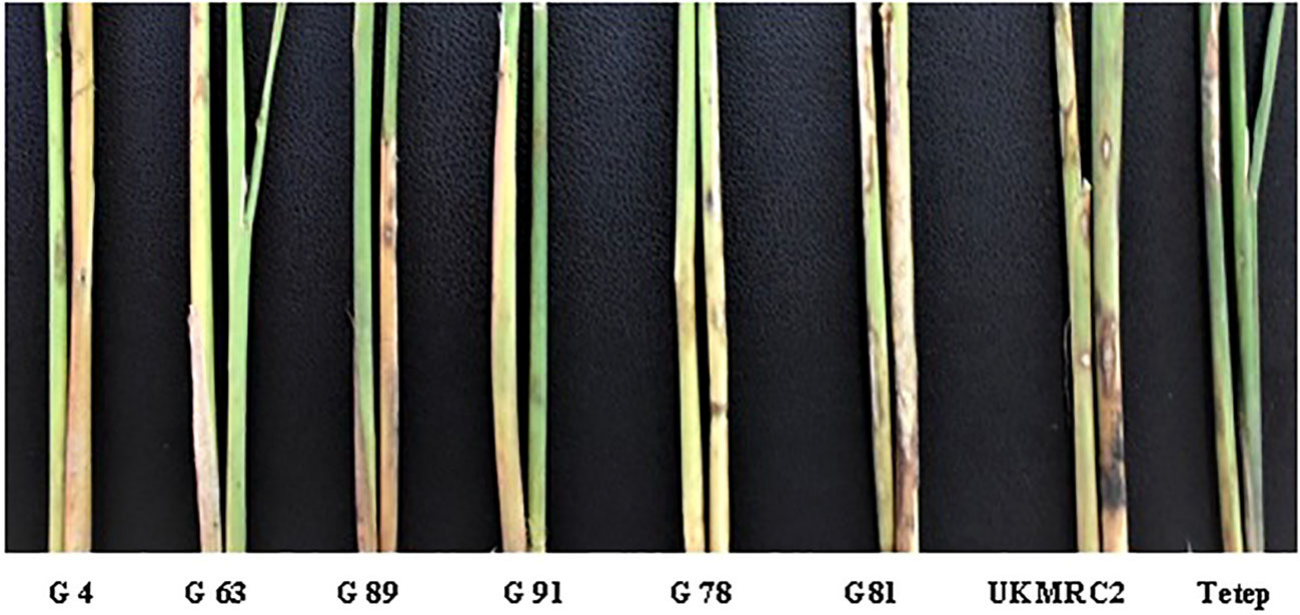
Sheath blight disease reactions of two parents and its six resistant progenies detected and phenotyped through artificial field inoculation of R. solani at UKM campus, Bangi, Selangor, Malaysia. The presence of resistance alleles are mentioned in the **Figure 7**.

Our field evaluation, subsequent QTL analyses, and gel-based detection the presence of QTLs in our developed BC_1_F_1_ progenies indicated that the *QTL qSBR11-1^TT^* was successful introgression in UKMRC 2 and it was verified by LOD value (3.25), and total phenotypic variance (14.6%) explained. The PAGE images confirmed the effect size of the QTLs. It was observed that the resistant progenies G78 and 81 (**Figure 7**) which contained the marker locus RM224 (**Figure 8**) showed a DSI score of 3.0 whereas the progenies G4, 63, 89, and 91 contained the markers locus RM7443 and RM27360 (**Figure 8**) and possessed a DSI score of 1.0. (**Figure 7**) These results indicated that these two flanking markers could be tightly linked with the *QTL qSBR11-1^TT^* and could be suitable for marker-assisted introgression into new elite background for SB resistance in the future. However, the QTL was fine mapped to a 0.85 Mb region on chromosome 11 with interval markers K39512 and sbq33 by Channamallikarjuna et al. (2010). The chromosomal location of the QTL was also harboring chitinase-III-like genes (Channamallikarjuna et al., 2010). ‘The Rice chromosome 11 and 12 sequencing consortium 2005’ reported that the most R-like genes and defense response-like genes (glucanases, chitinases, thaumatin-like proteins) are present in chromosome 11 as a large cluster of tandem arrays in positions, 30 to 40 cM, 80 to 90 cM, and 110 to 119 cM. Channamallikarjuna et al. (2010) reported a large cluster of 14 defense genes in which 11 chitinase genes are present at the 116.2 cM regions. The QTL *qSBR11-1^TT^* was identified in the same location as a major effect QTL by the same author. Singh et al. (2012) also introgressed the QTL *qSBR11-1^TT^* into ‘Pusa Basmati 1’ successfully by marker-assisted backcrossing.

## Conclusion

A couple of BC_1_F_1_ progenies resistant progenies were developed with a substantial amount of SB resistance in the UKMRC2 genetic background. Significant effects of the QTL *qSBR11-1^TT^* were detected with validation by QTL analysis with flanking and peak markers and phenotypic variation through field phenotyping procedures. The six SB-resistant genotypes are potential and could be advanced into the next generation for the selection of desirable breeding lines toward variety development. The donor alleles RM 7443 and RM 27360 were found to be tightly linked with SB QTLs *qSBR11-1^TT^* which might be a breeder choice for marker-assisted selection for further improvement of SB in modern popular rice varieties.

## Data availability statement

The original contributions presented in the study are included in the article/supplementary materials. Further inquiries can be directed to the corresponding author.

## Author contributions

MH: conducted the study and wrote the manuscript. MI: reviewed and made significant input in the discussion part of the manuscript. RS: reviewed the article and made contributions to the title and in the abstract. MB: coguide of the research and made the significant contributions to the manuscript.WR: supervised the overall study and contributed to the review and input in the manuscript. All authors contributed to the article and approved the submitted version.
